# Does the early social environment prepare individuals for the future? A match-mismatch experiment in female wild cavies

**DOI:** 10.1186/s12983-018-0261-1

**Published:** 2018-04-16

**Authors:** Susanne Sangenstedt, Carsten Szardenings, Norbert Sachser, Sylvia Kaiser

**Affiliations:** 1 Department of Behavioural Biology, University of Münster, Münster, Germany; 2 Münster Graduate School of Evolution, University of Münster, Münster, Germany; 3 Department of Psychology, University of Münster, Münster, Germany

**Keywords:** Prenatal, Female offspring, Adaptation, Behavioral development, Environmental matching, Energy demanding, Vigilance, Cortisol, Stress responsiveness, Wild cavy

## Abstract

**Background:**

The social environment that mothers experience during pregnancy and lactation has a strong effect on the developing offspring. Whether offspring can be adaptively shaped to match an environment that is similar to the maternal one is still a major question in research. Our previous work in wild cavies showed that females whose mothers lived in a stable social environment with few social challenges during pregnancy and lactation (SE-daughters) developed different behavioral phenotypes than females whose mothers lived in an unstable social environment with frequent social challenges during pregnancy and lactation (UE-daughters). In the present study we investigated whether SE-daughters are better adapted to a stable social environment, similar to their maternal one, than are UE-daughters, for which the stable social environment represents a mismatch with their maternal one. For this purpose, we established pairs of one UE- and one SE-daughter and housed them together under stable social conditions for one week. Dominance ranks, behavioral profiles, glucocorticoid levels, cortisol responsiveness and body weight changes were compared between the groups. We hypothesized that SE-daughters fare better in a stable social setting compared to UE-daughters.

**Results:**

After one week of cohabitation in the stable social condition, UE-daughters had higher glucocorticoid levels, tended to gain less body weight within the first three days and displayed higher frequencies of energy-demanding behaviors such as *rearing* and *digging* than SE-daughters. However, there was no difference in cortisol responsiveness as well as in dominance ranks between UE- and SE-daughters.

**Conclusion:**

Higher glucocorticoid levels and less body weight gain imply that UE-daughters had higher energy demands than SE-daughters. This high energy demand of UE-daughters is further indicated by the increased display of *rearing* and *digging* behavior. *Rearing* implies increased vigilance, which is far too energy demanding in a stable social condition but may confer an advantage in an unstable social environment. Hence, SE-daughters seem to better match a stable social environment, similar to their maternal one, than do UE-daughters, who encountered a mismatch to their maternal environment. This data supports the environmental matching hypothesis, stating that individuals manage the best in environments that correspond to their maternal ones.

## Background

Phenotypic plasticity is the capacity of an organism’s genotype to respond to environmental cues by changing the individual’s behavior, physiology or morphology [1–3]. These changes in the individual’s phenotype are mainly triggered by epigenetic processes and can be adaptive to prevailing environmental conditions [1, 2, 4, 5]. Especially in early life phases, environmental cues have a strong influence on the developing organism, whereby mothers are particularly important in mediating information about current environmental conditions [6–11]. As such, the environmental matching hypothesis assumes that according to the environmental information received in early life, individuals form an adaptive phenotype that not only has immediate benefits but also provides a fitness advantage in later life [12]. For example, small aquatic crustaceans develop specific morphological features when mothers are exposed to predator cues, and these features can be advantageous for predator defense later on [13].

Recently, the adaptive shaping of later life phenotypes has been investigated in terms of the early social environment that individuals experience [14–17]. Especially during the prenatal and early postnatal phase of life, the social environment can have a great impact on individuals’ behavior, neurophysiology and morphology [8, 11, 18–23]. The most consistent data on how the early social environment influences developing offspring is derived from domesticated guinea pigs (*Cavia aperea* f. *porcellus*) [8, 24]. Male guinea pigs whose mothers lived in an unstable social environment during pregnancy and lactation display an infantilized behavioral profile in later life (i.e. show juvenile-typical behavior) compared to male guinea pigs whose mothers lived in a stable social environment during pregnancy and lactation [25, 26]. In turn, female guinea pigs of mothers living in an unstable social environment during pregnancy and lactation show a behavioral and neuroendocrine masculinization (i.e. display male-typical behavior as well as increased plasma testosterone levels) in comparison to females of mothers from a stable social environment [27–29]. Concerning the question whether this phenomenon was brought up by domestication, the wild ancestors of guinea pigs, wild cavies (*Cavia aperea*), were tested in a similar experimental setup. This revealed comparable behavioral alterations in male as well as female offspring based on their early social environment [30, 31]. Hence, artificial selection during the process of domestication did not cause these phenotypic changes. Yet, the question arose whether an adaptive mechanism induces these phenotypic alterations.

In their natural habitat, wild cavies face different social environments [32, 33]. These depend on population densities that fluctuate due to changing predator pressures [32–35]. It was argued that in a high population density, wild cavies experience increased levels of aggression and competitive encounters over scarce resources. Further, social interaction partners frequently change, what can be defined as an unstable social environment [8]. In contrast, in a low population density, resources are likely sufficiently present and competition levels are probably low. Social interaction partners stay the same, as it can be found in a stable social environment [8]. According to the environmental matching hypothesis [12], wild cavies should have a higher fitness when facing environmental conditions in later life that are similar to their maternal ones. In this matter, a recent study found evidence that male wild cavies are better adjusted to a social environment that is similar to the one their mothers encountered during pregnancy and lactation [36]. However, whether also female wild cavies are better adapted to a social environment that is comparable to their maternal one is not yet clear.

In order to elucidate this possibility, the present study investigated whether females whose mothers lived in a stable social environment during pregnancy and lactation (SE-daughters) and females whose mothers lived in an unstable social environment during pregnancy and lactation (UE-daughters) show different behavioral and neuroendocrine reactions to a stable social environment. We established pairs, each containing one UE- and one SE-daughter, and housed them together under stable social conditions for one week. Regarding previous findings in males [36], we assumed the following in females: SE-daughters should be better adapted to the stable social setting, as it matches their early social environment, in comparison to UE-daughters, who encounter a mismatch. We screened dominance as an established proxy for fitness, as dominant females commonly have a higher reproductive success than subdominant ones [37–42]. Concerning this, we hypothesized that SE-daughters should be dominant over UE-daughters in a stable social setting. In addition, we expected that both groups should differ in their behavioral patterns, such as social orientation, sociopositive, courtship and sexual, play, attentive (i.e. vigilant) and digging behavior. As another proxy for fitness, we tested the activity and reactivity of the hypothalamic-pituitary-adrenal (HPA) axis in UE- and SE-daughters, because it is the major physiological system that enables vertebrates to adapt to challenging environmental conditions [43]. Since usually animals that are better adapted to a given environmental condition have lower HPA activity (but see [17]), we hypothesized that UE-daughters should have higher glucocorticoid levels and less body weight gain compared to SE-daughters when living together in a stable social setting. Furthermore, we assumed that after living together in a stable social condition for one week, UE-daughters should have higher cortisol (C) reaction values in a novel environment than SE-daughters.

## Methods

### Animals

The experiments were conducted with 22 female offspring of wild cavies of the species *Cavia aperea* ERXLEBEN, 1777, derived from a breeding stock established at the Department of Behavioural Biology, University of Münster. The animals were descendants from feral cavies trapped in the province of Buenos Aires, Argentina, in 1995 and from lineages belonging to the Universities of Bayreuth and Bielefeld, Germany. Since cavies have a uniform brown pelage, which does not allow for individual differentiation, they were marked by bleaching the fur with 32% hydrogen peroxide.

### General housing conditions

All animals were kept under the following standardized conditions: temperature about 22 °C, relative humidity about 50%, light/dark cycle 12:12 h with the light phase starting at 07:00 am. Commercial guinea pig diet (Höveler Meerschweinchenfutter 10700, Höveler Spezialfutterwerke GmbH & Co. KG, Dormagen, Germany, and Altromin 3023, Altromin Spezialfutter GmbH & Co. KG, Lage, Germany), hay and water were available ad libitum. This diet was supplemented with oat flakes weekly (Fortin Mühlenwerke GmbH & Co. KG, Düsseldorf, Germany). Vitamin C was added to the water twice per week. All animals were housed in wooden enclosures (height of the walls = 80 cm). The floors were covered with wood shavings for bedding (Allspan Olympia-Einstreu, Allspan GmbH, Karlsruhe, Germany) and cleaned every 4 weeks.

### Housing of pregnant and lactating females

Sixteen groups were composed, each consisting of one adult male and two adult females, because the basic social unit of wild cavies in nature is either a small harem or a pair [32, 33]. Groups were housed in 1.5 m^2^ enclosures that were enriched with a brick, two wooden branches and two cardboard boxes for shelter. Eight groups were held in a stable social environment while the other eight groups encountered an unstable social environment. Females experienced their assigned social environment throughout gestation, which lasts around 62 days [44]. Pups stayed with their mothers until weaning (age of 20 ± 1 days).

### Establishment of unstable and stable social environments

#### Unstable social environment (UE)

In the eight groups in the UE condition, one of the two females was transferred to the clockwise neighboring enclosure every second week. After a 1-week offset, the remaining female was rotated counter-clockwise in the same manner. Males remained in the enclosures. This regular exchange of females between different groups led to a change of group compositions once per week. Preweaning offspring were transferred together with lactating females.

#### Stable social environment (SE)

In contrast to UE-groups, the composition of the eight SE-groups remained constant throughout the study. To prevent handling bias, all females and their preweaning offspring were handled in the same manner as UE-females at corresponding times.

### Housing of daughters

The experiment was conducted with 22 daughters of females that had at least one previous litter in the UE- and SE-groups (daughters of mothers living in an unstable social environment during pregnancy and lactation (UE-daughters): N = 11; daughters of mothers living in a stable social environment during pregnancy and lactation (SE-daughters): N = 11). Subjects were separated from their maternal groups after weaning (day 20 ± 1 of age) because females can get pregnant at a very early age (approx. 30 days of age) [45]. They were transferred to a 0.5 m^2^ wooden enclosure, which was supplemented with two wooden houses, each resembling a tent with two triangle sides and one opening (21.5 × 23 × 12 cm). Subjects joined another daughter from the same social environment and of about the same age, so that one UE-daughter was housed together with another UE-daughter and one SE-daughter with another SE-daughter (max. age difference = 10 days). Pair mates originated from different natal groups. They were unrelated and unfamiliar with each other.

**Fig. 1:**
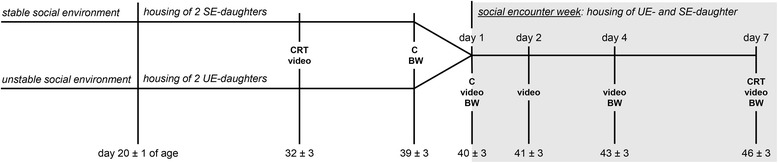
Experimental design. UE-daughters: daughters of mothers living in an unstable social environment during pregnancy and lactation; SE-daughters: daughters of mothers living in a stable social environment during pregnancy and lactation. After weaning (day 20 ± 1 of age) UE-daughters as well as SE-daughters were held in pairs with an unfamiliar female of about the same age and from the same early environmental condition. On day 40 ± 3 of age, one UE-daughter and one SE-daughter were placed together in a new enclosure and stayed there for seven days, which was labelled the social encounter week. Spontaneous behavior (video) of UE-daughter/SE-daughter pairs was recorded around day 32 ± 3 of age; spontaneous behavior of UE- and SE-daughters was recorded on the first, second, fourth and last day of the social encounter week. Blood samples were taken one day before and on the first day of the social encounter week in order to determine plasma cortisol concentrations (C). A Cortisol Response Test (CRT) was performed one week prior to and on the last day of the social encounter week. Body weight (BW) was measured after each blood sampling as well as on the fourth day of the social encounter week

After staying together for about three weeks, these pairs were separated, and one UE-daughter and one SE-daughter (mean age ± SEM: 40 ± 3 days) were transferred to a new enclosure where they were housed together for seven days (labelled as “social encounter week”; see Fig. 1). A previous study found significant differences in behavioral patterns, such as play and agonistic behavior, between UE- and SE-daughters at exactly this age [31]. Further, seven days were scheduled as experimental time because pilot studies showed that wild cavies already display major adaptations and clear dominance relations after living together for 1-2 days.

The social encounter week started at 09:00 am (± 15 min) when the UE- and SE-daughters were simultaneously put in a 1 m^2^ wooden enclosure that contained one house as hiding place. Subjects were age-matched so that the two testing partners did not differ by more than seven days. Furthermore, equal numbers of dominant and subdominant UE- and SE-daughters were chosen and assigned to their pairs in a randomized order (i.e. pairs of dominant-dominant, dominant-subdominant, subdominant-subdominant were placed together). To determine dominance ranks of subjects before the social encounter week, UE- and SE-daughters were videotaped for 2-5 h one week after they were housed together with a female of the same early social environment (see Fig. 1; for more details see *Measurement of behavioral profiles and dominance ranks*).

### Measurement of behavioral profiles and dominance ranks

During the social encounter week, UE- and SE-daughters were videotaped on the first and second day for 2 h and on the fourth and seventh day for 1 h in order to analyze behavioral profiles as well as dominance relations (see Fig. 1). Presumably, subjects should show more behavioral activity during the initial days of the social encounter week, as they were getting accustomed to their new social partner and exploring their new environment. Social orientation, sociopositive, courtship and sexual, agonistic, play, attentive and digging behavior of UE- and SE-daughters were examined in order to assess behavioral profiles. The definitions and categorization of behaviors were based on previous work by [30, 31, 40], and are listed in Table 1. Recording always took place between 9:00 and 11:00 h (± 15 min). The first 15 min of recordings were omitted in order to minimize the effect of disturbance by the experimenter. On the first day of the social encounter week, the first 15 min of the recording were however included into the analysis in order to capture initial behavioral reactions of subjects towards the novel situation. All behaviors were recorded using continuous recording and focal animal sampling [46].

**Table 1:** Behavioral elements, listed in their behavioral systems, and their definitions

Behavioral element	Definition
Social orientation behavior:	
*Naso-nasal sniffing*	The focal animal moves its nose towards the nasal region of another animal and sniffs, licks and/or nuzzles. The distance is less than one snout-width.
*Naso-anal sniffing*	The focal animal moves its nose towards the ano-genital region of another animal and sniffs, licks and/or nuzzles. The distance is less than one snout-width.
Sociopositive behavior:	
*Resting with bodily contact*	Two animals are sitting or lying side by side and none of them shows any movements for at least 5 s. The nearest parts of the animals’ bodies have direct contact. The behavior stops if one of them moves continuously for more than 5 s.
Courtship & sexual behavior:	
*Rumba*	The focal animal moves slowly towards another animal and steps rhythmically from one hind leg to the other. The head can be lowered and held parallel to the ground. The body of the focal animal can show a curve when it approaches the other animal. Rumba can also be shown without a forward movement but a continuous stepping in place of the hind legs with a shift from one hind leg to the other.
*Mounting*	The focal animal moves the upper part of its body onto another animal’s back from behind.
Agonistic behavior:	
*Fixation*	The focal animal actively turns its head towards the other one. This does not result from the current general body movement.
*Head-up*	The distance between the animals is less than one body length. The focal animal lifts its mouth quickly upwards.
*Chase*	The focal animal follows another animal over a distance of at least one body length. This happens with high velocity. During this interaction, the distance between both animals never exceeds two body lengths. Chasing is terminated if the distance between the animals exceeds two body lengths for more than 3 s.
*Retreat*	The focal animal increases its distance to another animal to more than one body length. This happens either after an interaction of the animals or after an approach by the other animal.
*Head-thrust/bite*	The focal animal jabs its head quickly towards the other one. The other animal can be touched by this movement. The head is usually directed forward, but the animal may also direct the head sideways.
*Curved body posture*	The focal animal moves its head as well as its hindquarters towards the other one. The whole body shows a bended line. Both animals are oriented sideways to each other.
*Attack-lunge*	The focal animal quickly jumps towards another animal with its whole body. Both animals may display this behavior simultaneously. This movement may lead to bodily contact.
*Brawl*	Two animals scuffle with one another and try to twist the opponent to its side or onto its back. The animals may also try to bite one another. The behavior is finished if there is no longer body contact between the two.
Play behavior:	
*Frisky hops*	The focal animal makes one or a series of upward leaps and turns the head or foreparts sharply while in air.
*Run off*	The focal animal starts with a short and fast run, then suddenly stops and changes the direction.
Attentive behavior:	
*Rearing*	The focal animal lifts the fore part of its body so that the forelegs do not touch the floor anymore. It may touch the walls of the enclosure with its forelegs.
Other:	
*Digging*	The focal animal moves its forepaws jerkily over the bedding and may in consequence shift bedding. The behavior stops when the focal animal does not show it again within 3 s.

All behavioral parameters were scored as frequencies with the exception of *resting with bodily contact*, which was recorded as duration. Definitions were based on previous work by [30, 31, 44]

Dominance relations were calculated based on recorded frequencies of *retreat* (see Table 1), because this behavioral pattern is the most reliable indicator of subdominance in guinea pigs [47]*.* Following [48], the subject’s rank was determined by means of an index based on the ratio between the number of agonistic encounters that caused a retreat of the partner animal (*Ag*^+^) divided by all agonistic encounters that caused a retreat of the partner animal (*Ag*^+^) as well as of the subject (*Ag*^−^):

$$ \frac{Ag^{+}}{Ag^{+}+{Ag}^{-}} $$

The index varied from 0 to 1. The higher the index, the higher ranking the subject. Dominance relations were considered clear when rank indices differed by more than 0.5.

### Measurement of cortisol values

In order to determine plasma C concentrations of UE- and SE-daughters in reaction to the new social environment, blood samples were taken one day before as well as 4 h after onset of the social encounter week (see Fig. 1). Pilot studies revealed that wild cavies strongly react to the introduction of a new social partner. Further, they still show elevated C values 4 h after being transferred to a new environment [49].

Blood samples were taken at 13:00 h (± 15 min) to additionally control for possible influences of circadian rhythm on hormone concentrations, as the domestic form of the wild cavy, the guinea pig, shows diurnal variations in plasma C titers with a peak around this time of the day [50, 51]. The housing room of the experimental animals was not disturbed 2 h prior to blood sampling. After blood sampling, subjects were returned to their enclosures.

### Cortisol Response Test

Animals were further tested using a Cortisol Response Test (CRT) seven days before as well as on the last day of the social encounter week in order to evaluate their general hormonal responsiveness towards a novel physical environment (see Fig. 1). During the CRT the subjects’ baseline C value and C response during a 2 h exposure to an unfamiliar environment, with no other conspecific and no shelter, were measured [52, 53]. A novel environment has been shown to act as a psychological stressor in guinea pigs and cavies, causing an increase in C levels [54, 55]. The two CRTs were exactly two weeks apart and started at 13:00 h (± 15 min). At the beginning of each test, the subject was caught in its home enclosure and blood samples were taken. Subsequently, the animal was transferred to a different room of wild cavy husbandry and placed in a testing enclosure (100 × 100 × 80 cm), which was covered with fresh wood shavings and contained water and food pellets ad libitum. After 60 and 120 min, another blood sample was taken. When the last blood sampling was done, the testing subject was returned to its home enclosure. C levels measured at the beginning of the test represent baseline values (*C0*) while all other C levels describe reaction values (*C1* = reaction C value after 1 h; *C2* = reaction C value after 2 h).

### Measurement of body weight

Body weight was recorded after each blood sampling as well as on the fourth day of the social encounter week (see Fig. 1). The absolute change in body weight was calculated during the social encounter week (absolute change from day 1 to day 4 as well as from day 4 to day 7 of the social encounter week).

### Blood sampling

Blood samples were collected from the blood vessels of the ears. A muscle salve (Finalgon® Salbe, Boehringer Ingelheim Pharma GmbH & Co. KG, Ingelheim am Rhein, Germany) was applied to stimulate circulation in the ears. After removing the salve with a tissue, vessels were illuminated with a small LED light and pricked with a sterile injection needle. About 0.1 ml of blood was collected in heparinized capillary tubes. Samples for determination of C levels were taken within the first 3 min after entering the room, as plasma C levels do not increase significantly within the first 5 min after disturbing the room and hence the procedure reliably measured current plasma C levels before entering the room. This blood sampling method is a non-stressful procedure for the animals and does not elicit significant struggling [56]. After the blood sampling, plasma was immediately separated by centrifugation (13,000 rpm for 7 min) and deep frozen (− 20 °C) until analysis.

### Endocrine analyses

Plasma cortisol levels were analyzed in duplicate using a Cortisol Luminescence Immunoassay kit (ELISA; Cortisol ELISA Kit, IBL International GmbH, Hamburg, Germany). The antibody used cross-reacted with relevant steroids as follows: cortisol 100%, prednisolone 29.8%, 11-desoxycortisol 8.48%, cortisone 4.49%, prednisone 2.12%, corticosterone 1.99%, 6β-hydroxycortisol 1.03%. The intra-assay coefficient of variation was 3.2%; the inter-assay coefficient of variation was 6.1%.

### Data analysis and statistics

Recorded videos were evaluated using Interact 9.7.4.5 (Mangold International GmbH, Arnstorf, Germany). It was not possible to exclude the influence of partner animals on all measured variables, thus data taken during the social encounter week were considered dependent. Data were hence analyzed with a paired samples Wilcoxon signed-rank test for pairwise comparisons and with a Friedman test for repeated measures unless normality assumptions were met, in which case paired samples t-tests or linear models were used [57]. Statistical analysis was performed in the R environment [58], whereby linear mixed-effects models were calculated using the lme4 package [59] with fixed factors for early social environment, day of testing and time. We further computed Pearson correlations in order to test whether subjects who were dominant in their groups before the social encounter week were again dominant during the social encounter week. Whether dominant and subdominant subjects differed in their baseline C levels and body weights was checked by applying independent t-test or Mann-Whitney U test [57]. Behavioral elements were combined in their respective behavioral systems and analyzed, except for sociopositive behavior, which was shown so rarely that it had to be excluded from statistical analyses. All results are based on a sample size N_UE-daughters_ = N_SE-daughters_ = 11 unless otherwise stated. Differences of α ≤ 0.05 were considered significant. To account for multiple comparisons of repeated measures, the Bonferroni correction was applied. We report raw *p*-values but indicate statistical significance based on corrected α-levels.

Graphs were created using Sigma Plot 12.5 for Windows (SPSS, Inc., Chicago, IL, USA).

## Results

### Behavior

**Fig. 2:**
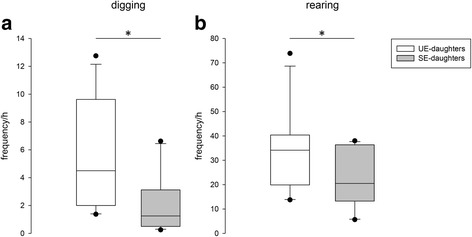
Frequency of *digging* (**a**) and *rearing* (**b**) per hour of UE- and SE-daughters during the social encounter week. Spontaneous behavior was merged from recorded videos on the first, second, fourth and last day of the social encounter week. UE-daughters: daughters whose mothers had lived in an unstable social environment during pregnancy and lactation; SE-daughters: daughters whose mothers had lived in a stable social environment during pregnancy and lactation. Data are shown as medians, 10th, 25th, 75th and 90th percentiles, and outliers. Statistics: Wilcoxon signed-rank test, *p < 0.05. N_UE-daughters_ = N_SE-daughters_ = 11

Daughters of mothers who lived in an unstable social environment during pregnancy and lactation showed higher frequencies of *digging* (Wilcoxon, W = 62, *p* = 0.011; see Fig. 2a) and *rearing* (Wilcoxon, W = 56.5, *p* = 0.041; see Fig. 2b) than daughters of mothers who lived in a stable social environment during pregnancy and lactation. All other behaviors showed no differences between the groups (see Table 2).

**Table 2:** Behavioral elements (frequencies/h) of UE- and SE-daughters during the social encounter week

	UE-daughters	SE-daughters	Wilcoxon signed-rank test	
			W =	p =
Social orientation behavior	0.5 [*0 – 2.5*]	0.75 [*0 – 2.13*]	16	0.833
Sociopositive behavior	0 [*0 – 335.08*]^†^	0 [*0 – 335.08*]^†^	–	–
Courtship & sexual behavior	0 [*0 – 0.63*]	0 [*0 – 0.13*]	4.5	0.586
Agonistic behavior	24.13 [*17.75 – 45.25*]	28.25 [*17.38 – 47.13*]	33	0.999
Play behavior	0.5 [*0 – 10.63*]	1.13 [*0 – 6.13*]	32.5	0.646

Spontaneous behavior was merged from recorded videos on the first, second, fourth and last day of the social encounter week. UE-daughters: daughters whose mothers had lived in an unstable social environment during pregnancy and lactation; SE-daughters: daughters whose mothers had lived in a stable social environment during pregnancy and lactation. Values are given as medians; numbers in brackets give minimum and maximum values. Sociopositive behavior is given as ^†^duration (s/h); it was shown so rarely that it had to be excluded from statistical analysis. Statistics: Wilcoxon signed-rank test. N_UE-daughters_ = N_SE-daughters_ = 11

During the time when UE- and SE-daughters lived in pairs with a female of the same early social environment, they formed clear dominance relations (data not shown). These rank indices measured before the social encounter week did not predict later rank indices during the social encounter week (Pearson correlation, *t* = − 0.56, *p* = 0.580). During the social encounter week, six UE-daughters and four SE-daughters were dominant while dominance relations of one pair remained unclear. Accordingly, there was no difference in whether UE- or SE-daughters became dominant (Wilcoxon, W = 36, *p* = 0.618; see Table 3).

**Table 3:** Frequency of retreat per hour and indices of dominance of UE- and SE-daughters during the social encounter week

Group	retreat/h		index of dominance	
	*UE-daughters*	*SE-daughters*	*UE-daughters*	*SE-daughters*
1	4.17	36.67	**0.90**	0.10
2	24	1.5	0.06	**0.94**
3	8.67	32.5	**0.79**	0.21
4	35.83	1.67	0.04	**0.96**
5	8.33	48.33	**0.85**	0.15
6	3.83	20.67	**0.84**	0.16
7	59	14.17	0.19	**0.81**
8	19	16.67	0.47	0.53
9	16.5	4.33	0.21	**0.79**
10	5.33	21.17	**0.80**	0.20
11	7.67	46.5	**0.86**	0.14

Rank indices range from 0 to 1. The higher the index, the higher ranked the subject. Spontaneous behavior was merged from recorded videos on the first, second, fourth and last day of the social encounter week. UE-daughters: daughters whose mothers had lived in an unstable social environment during pregnancy and lactation; SE-daughters: daughters whose mothers had lived in a stable social environment during pregnancy and lactation. Data are given as original values. Indices of dominant subjects are shown in bold. Statistics: Wilcoxon signed-rank test, n.s. N_UE-daughters_ = N_SE-daughters_ = 11

### Cortisol

**Fig. 3:**
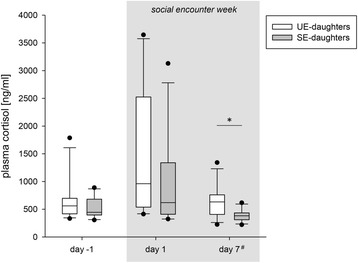
Plasma cortisol concentrations (ng/ml) of UE- and SE-daughters one day before, on the first day and on the last day of the social encounter week. ^**#**^Plasma C values on the last day of the social encounter week are baseline values of the CRT performed on that day. UE-daughters: daughters whose mothers had lived in an unstable social environment during pregnancy and lactation; SE-daughters: daughters whose mothers had lived in a stable social environment during pregnancy and lactation. Data are shown as medians, 10th, 25th, 75th and 90th percentiles, and outliers. Statistics: Wilcoxon signed-rank test, Bonferroni corrected, *p ≤ 0.010. N_UE-daughters_ = 11; N_SE-daughters_ = 10-11

One day before the start of the social encounter week, UE- and SE-daughters did not differ in C concentrations (Wilcoxon, W = 38, N_UE-daughters_ = N_SE-daughters_ = 10, *p* = 0.322). Four hours after being introduced to each other, C values were increased in both UE- and SE-daughters (up to 121% in UE-daughters and 88% in SE-daughters), whereby this increase was not significant (Friedman test, UE-daughters: χ^2^(2) = 3.46, *p* = 0.178; SE-daughters: χ^2^(2) = 2.36, *p* = 0.307). In addition, increased C levels did not significantly differ between groups (Wilcoxon, W = 48, *p* = 0.206). However, six days later, when C concentrations dropped down again, UE-daughters had significantly higher C values than SE-daughters (Wilcoxon, W = 61, *p* = 0.010; see Fig. 3).

When comparing C values of dominant and subdominant animals, there were no differences found on any day (Mann-Whitney U, *day − 1:* U = 40, N_dom_ = 9, N_sub_ = 10, *p* = 0.720; *day 1:* U = 65, N_dom_ = N_sub_ = 10, *p* = 0.280; *day 7:* U = 43, N_dom_ = N_sub_ = 10, *p* = 0.631).

Analysis of plasma C concentrations in the Cortisol Response Test, which was performed twice (one week prior to the social encounter week and on the last day of the social encounter week), revealed that C values of UE- and SE-daughters significantly increased in both tests (LMM, F = 4.34, *p* = 0.016). When comparing both groups, we found no differences in their C responsiveness (LMM, F = 0.13, p = 0.720). Also, C values did not differ between the two testing days (LMM, F = 0.63, *p* = 0.429). Overall, there was no interaction effect between time, early social environment and day of testing on C levels (LMM, F = 0.11, *p* = 0.894; see Table 4).

**Table 4:** Plasma cortisol concentrations (ng/ml) of UE- and SE-daughters during the Cortisol Response Test (CRT) one week prior to and on the last day of the social encounter week

	one week prior to social encounter week		last day of social encounter week		LMM							
					time		early social environment		day of testing		time × early social environment × day of testing	
	*UE-daughters*	*SE-daughters*	*UE-daughters*	*SE-daughters*	F =	p =	F =	p =	F =	p =	F =	p =
plasma cortisol concentrations during CRT (ng/ml)												
C0	667.1 ± 90.0	554.9 ± 106.2	635.8 ± 88.8	389.9 ± 32.4	4.34	0.016	0.13	0.720	0.63	0.429	0.11	0.894
C1	3610.6 ± 281.7	3634.8 ± 344.3	3741.2 ± 410.7	3446.5 ± 292.9								
C2	4185.2 ± 440.1	4355.3 ± 385.9	4525.4 ± 478.2	4972.2 ± 298.6								

The test was performed twice, exactly two weeks apart. UE-daughters: daughters whose mothers had lived in an unstable social environment during pregnancy and lactation; SE-daughters: daughters whose mothers had lived in a stable social environment during pregnancy and lactation. Data are given as untransformed mean values ± SEM. Statistics: LMM. N_UE-daughters_ = 10-11; N_SE-daughters_ = 9-11

### Body weight

**Fig. 4:**
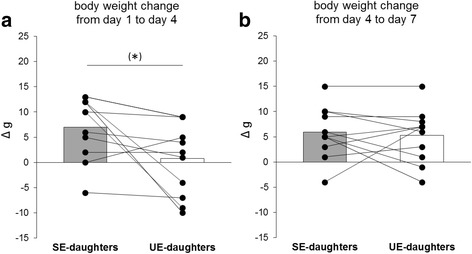
Body weight change (Δg) of UE- and SE-daughters from the first day to the fourth day (**a**) and from the fourth to the last day (**b**) of the social encounter week. UE-daughters: daughters whose mothers had lived in an unstable social environment during pregnancy and lactation; SE-daughters: daughters whose mothers had lived in a stable social environment during pregnancy and lactation. Data are shown as means with single data points connecting partner animals. Statistics: Wilcoxon signed-rank test, significances indicated by ^(^*^)^ were only true before Bonferroni correction. N_UE-daughters_ = N_SE-daughters_ = 11

UE- and SE-daughters did not differ in body weights before they entered the social encounter week (mean body weight ± SEM: UE-daughters = 252.2 ± 6.8 g; SE-daughters = 251.3 ± 9.0 g; paired samples t-test, t(10) = 0.12, *p* = 0.906). Concerning the absolute change in body weight, SE-daughters gained more weight than UE-daughters from the first to the fourth day of the social encounter week (Wilcoxon, W = 7, *p* = 0.041; see Fig. 4a), but this difference was not statistically significant after Bonferroni correction. From the fourth to the last day of the social encounter week, there was no difference in body weight change between UE- and SE-daughters (Wilcoxon, W = 17.5, *p* = 0.592; see Fig. 4b).

When comparing body weights between dominant and subdominant animals, no differences were found on any day (independent t-test, N_dom_ = N_sub_ = 10, *day − 1:* t(18) = 1.17, *p* = 0.256; *day 1:* t(18) = 1.35, *p* = 0.194; *day 4:* t(18) = 1.48, *p* = 0.156; *day 7:* t(18) = 1.48, *p* = 0.157).

### Discussion

The present study investigated whether female wild cavies whose mothers lived in a stable social environment during pregnancy and lactation (SE-daughters) are better adapted to a similar stable social setting in later life compared to females whose mothers lived in an unstable social environment during pregnancy and lactation (UE-daughters). For this purpose, we provided a stable social condition that represented a match to the early social environment of SE-daughters and a mismatch to the early social environment of UE-daughters. After one week of cohabitation in the stable social condition, there was no difference in whether UE-daughters or SE-daughters became dominant. Surprisingly, we did not find a behavioral masculinization (i.e. display of male-typical behavior) of UE-daughters compared to SE-daughters, as it was described before [31]. Presently, we have no plausible explanation for this. However, we found that, in comparison to SE-daughters, UE-daughters displayed higher frequencies of energy-demanding behaviors such as *rearing* and *digging* and had higher glucocorticoid levels after one week in the stable social setting.

### An adaptive shaping of neuroendocrine and behavioral profiles to the early social environment?

One day before as well as on the first day of the social encounter week, UE- and SE-daughters did not differ in their glucocorticoid levels. Further, 4 h after being transferred to the stable social condition together, C levels of UE- and SE-daughters tended to be elevated, although this increase was not statistically significant. Also, because UE- and SE-daughters did not differ in their general C responsiveness when tested in a Cortisol Response Test, we conclude that female wild cavies have similar acute C reactions to a new (social) environment, irrespective of their early social environment.

While UE- and SE-daughters showed no differences in C values at the beginning of the social encounter week, they strikingly differed in their glucocorticoid levels in the long term. On the last day of the social encounter week, C values of UE- and SE-daughters had declined back to pretesting levels, yet remarkably, C levels of UE-daughters were significantly higher than of SE-daughters. This indicates that UE-daughters had elevated activity in their HPA axis after living together with SE-daughters for a week. The HPA axis is activated in order to provide organisms with additional energy so that they can appropriately adjust themselves to challenging situations [41, 60, 61]. Higher HPA activity in UE-daughters thus shows that they had an increased need for energy in the stable social condition compared to SE-daughters [62, 63]. This is also reflected in the observation that UE-daughters tended to gain less body weight compared to SE-daughters within the first three days of the social encounter week.

Higher energy mobilization in UE-daughters than in SE-daughters could be an adaptation to the unstable social environment, in which UE-mothers lived during pregnancy and lactation. There is good evidence that an unstable social environment is highly unpredictable and consists of frequent social challenges, which is likely to be found in high-density populations in the wild [8]. This condition can be highly energy demanding for individuals, as it requires them to cope with a variety of stressors, what is often related to increased activity of specific behaviors [64, 65]. Consequently, we found higher levels of the energy-demanding behavioral patterns *digging* and *rearing* in UE-daughters than in SE-daughters during the social encounter week. In particular, *rearing* is associated with attentiveness, as it was previously described in wild cavies [30, 55], and it may indicate increased vigilance in UE-daughters, which has been suggested to be adaptive in adverse environments [66–69]. In the wild, a higher vigilance might be advantageous for UE-daughters, as it enhances their chances of detecting approaching predators, which are naturally attracted to high population densities of prey [70]. In comparison, in a low-density population (i.e. a stable social environment), it seems likely that competition levels as well as the frequency of predatory threats are lower. There, an increased vigilance is not needed and is far too energy demanding. Thus, UE-daughters unnecessarily mobilized energy for being active during the social encounter week, which makes them less well adapted to the stable social condition than SE-daughters.

What is the underlying mechanism behind these findings? On the one hand, it could be a maternal manipulation of the offspring’s development during the pre- and/or early postnatal phase, also known as maternal effects [10]. During the prenatal phase the maternal perception of current environmental conditions can be mediated by hormones, which are transmitted to the offspring across the placenta [1, 10, 71]. These hormones can affect the organizational pathways of the offspring’s developing brain, causing physiological, neuroendocrine and behavioral changes in the offspring’s phenotype [22, 72, 73]. Maternal effects can further be mediated by maternal care during the lactation period, resulting in long-lasting changes in the offspring’s behavioral and neuroendocrine systems [74–76]. On the other hand, it is also possible that the offspring’s own perception of environmental conditions shortly after birth persistently shape phenotypic traits, independent from the mother’s perception of environmental conditions [2, 3] .

In wild cavies, it is most likely that endocrine signals of the mother shape the offspring’s phenotype during pregnancy [11]. Wild cavies are a precocial species with a relatively long gestation, during which most neural and other development occurs [44]. This makes it a conducive time for maternal hormones to shape the behavioral development of offspring [10, 11]. Pups are already highly developed at birth and require little maternal care, which limits the chance of a maternal shaping after birth [11]. In addition, studies in guinea pigs underlined that the behavioral masculinization of female offspring is exclusively administered during the prenatal phase, i.e. by maternal hormones during gestation [26, 29, 77]. Conclusively, regarding our results, it may be that SE-daughters have been adaptively shaped by their mothers to fit in a stable social environment, which is highlighted by their lower HPA activity and less frequent displays of energy-demanding behavioral patterns than was observed for UE-daughters in a stable social setting. In the case of UE-daughters, it is possible that UE-mothers prepared their daughters through maternal effects to meet the challenges of an unstable social environment by adjusting their HPA axis and behavioral activity.

Yet, whether offspring are adaptively shaped by early environmental cues in order to match similar environmental conditions in later life can only be confirmed by a full factorial design approach [78]. With the existing data, we cannot exclude the possibility that SE-daughters have a general advantage over UE-daughters due to their beneficial early environment, as proposed by the silver spoon hypothesis [79]. In this regard, the silver spoon hypothesis states that individuals born in “poor” environmental conditions have a life-long disadvantage compared to individuals born in “good” conditions [79]. However, studies on male offspring do not support the silver spoon hypothesis but rather underline that an adaptive shaping to the early social environment in wild cavies exists [36]. Thus, we favor the assumption that female wild cavies show an environmental matching effect, where individuals manage the best in a social environment that corresponds to the one their mothers encountered during pregnancy and lactation.

## Conclusions

Although dominance profiles and other behaviors did not differ between UE- and SE-daughters, we found other good indications that UE-daughters are not as well adapted as SE-daughters to living in a stable social environment in later life. Additional studies are needed to confirm that UE-daughters are, in turn, better adapted than SE-daughters to living in an unstable social environment. So far, our findings suggest that offspring can be adaptively shaped to match an environment that is similar to the maternal one. Regarding this, it seems likely that maternal effects play a role in shaping the offspring to match a social environment that resembles the one the mothers encountered during pregnancy and lactation.
